# Supplementary material for: Content-rich biological network constructed by mining PubMed abstracts
Source: BMC Bioinformatics. 2004 Oct 8;5:147. doi: 10.1186/1471-2105-5-147 (PMC528731; doi:10.1186/1471-2105-5-147)
Supplement: Additional File 2 — The original results of the above study (non-essential files are deleted to keep the file size under the limit set by BMC bioinformatics). [file 1471-2105-5-147-S2.bz2 › chilibotAdditionalFile2/dip10/303ID9501253E1300/html/APP_A2M.html]

 


 **APP** and **A2M** 
  
Found 50 abstracts in PubMed, retrieved 10.  
 

 What does Google say? 
 PDF only 
| .edu only 

---

**Interactive relationship** (e.g. stimulation, inhibition, etc)

**Neutral relationship**- Haptoglobin Hp and alpha 2 macroglobulin  [ **A2M** ]  alpha 2 M  [ **A2M** ]  are among the most important  **APP**  in rats.  Ref: 11521461 Vojnosanit Pregl< MedlineTA>Vojnosanit Pregl,

**Non-interactive relationship** (e.g. studied together, co-existance, homology, etc.)

- Alpha 2 macroglobulin  [ **A2M** ]  mediated degradation of amyloid beta  [ **APP** ]  1 42 a mechanism to enhance amyloid beta  [ **APP** ]  catabolism.  Ref: 11161627 Exp Neurol, 2001
- The responses of liver glucocorticoid receptor GR and genes coding for a glucocorticoid inducible tyrosine aminotransferase TAT and two acute phase proteins  **APP**  alpha2 macroglobulin alpha2  [ **A2M** ]  M and gamma fibrinogen Fb to changes in glucocorticoid GC and proinflammatory AP cytokine contents have been examined in rats after single or combined treatments with turpentine oil, dexamethasone Dex and adrenalectomy.  Ref: 11490095 Biol Signals Recept< MedlineTA>Biol Signals Recept, 2001
- As for the three early onset genes the amyloid beta  [ **APP** ]  protein precursor and the two presenilins and for the other late onset gene ApoE, DNA polymorphisms in the  **A2M**  gene associated with AD result in significantly increased accumulation of amyloid plaques in AD brains.  Ref: 10959035 Exp Gerontol, 2000
- Most of these studies have examined only one or two polymorphisms in a defined population, which provides a lack of a general view of the Alzheimer s disease genetic component.OBJECTIVETo overcome this limitation, nine polymorphisms in seven different candidate genes  **A2M** , ACT, APOE,  **APP** , BH, HSP70 2, and IL1 A were genotyped.METHODSThe sample comprised 112 Alzheimer s disease patients and 89 controls from Spain.  Ref: 12782964 Psychiatr Genet, 2003
- While rare mutations in the early onset genes amyloid precursor protein, and presenilins 1 and 2 lead to increased generation of specific forms of the amyloid beta  [ **APP** ]  protein A, beta, common polymorphisms in the late onset genes apolipoprotein E and alpha 2 macroglobulin  [ **A2M** ]  are thought to alter the clearance and degradation of A, beta in brain.  Ref: 11464541 Rev Med Chil, 2001
